# Supplementary material for: Construction of Porcine Epidemic Diarrhea Virus-Like Particles and Its Immunogenicity in Mice
Source: Vaccines (Basel). 2021 Apr 11;9(4):370. doi: 10.3390/vaccines9040370 (PMC8069460; doi:10.3390/vaccines9040370)
Supplement: Supplementary file 1 [file vaccines-09-00370-s001.pdf]

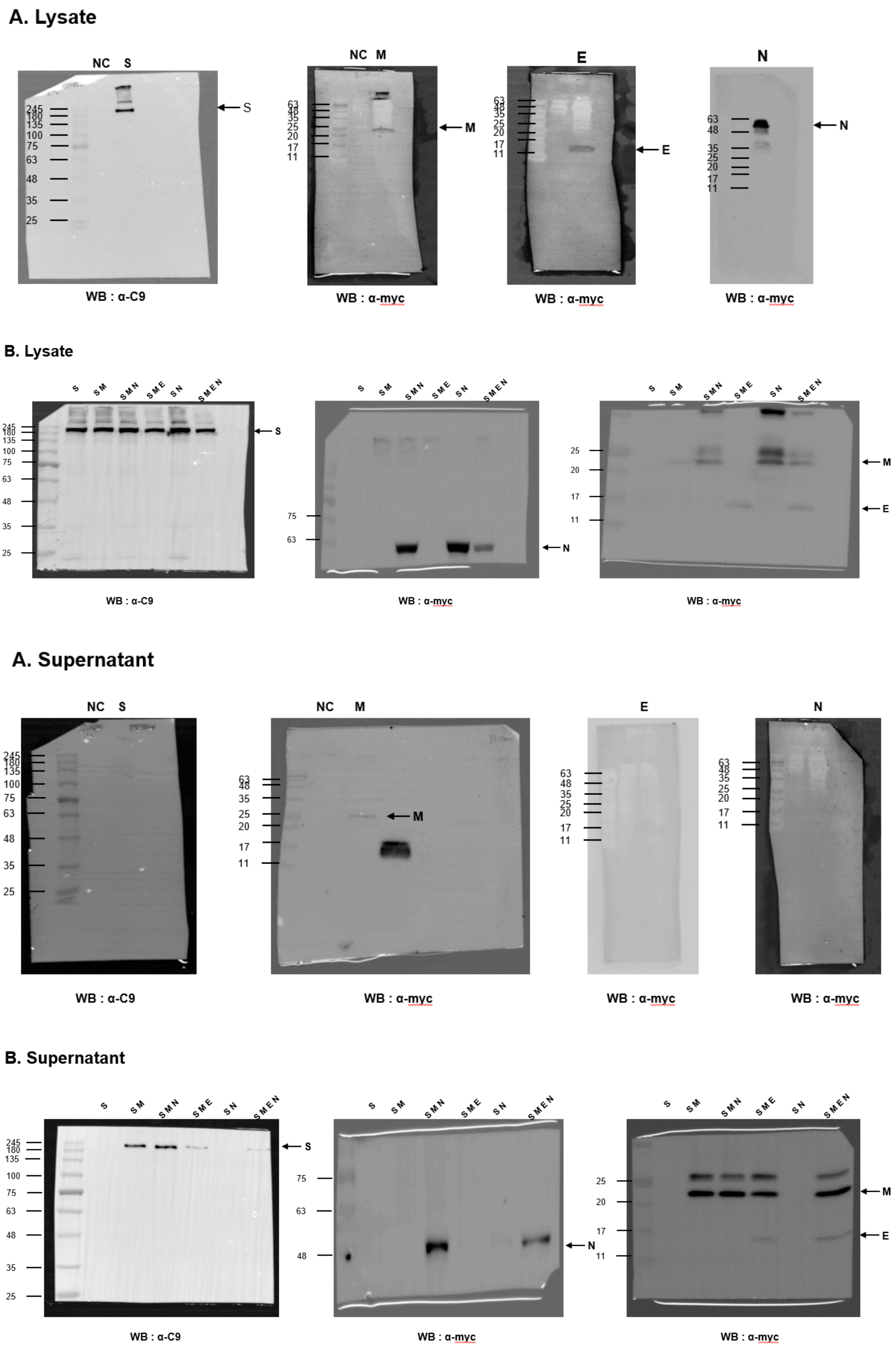

Figure S1. Generation of PED VLPs in mammalian expression system.
